# Rapid Resolution Liquid Chromatography Coupled with Quadrupole Time-of-Flight Mass Spectrometry-Based Metabolomics Approach to Study the Effects of Jieduquyuziyin Prescription on Systemic Lupus Erythematosus

**DOI:** 10.1371/journal.pone.0088223

**Published:** 2014-02-05

**Authors:** Xinghong Ding, Jinbo Hu, Chengping Wen, Zhishan Ding, Li Yao, Yongsheng Fan

**Affiliations:** 1 Analysis and Testing Center, Zhejiang Chinese Medical University, Hangzhou, China; 2 College of Pharmaceutical Science, Zhejiang Chinese Medical University, Hangzhou, China; 3 College of Basic Medicine, Zhejiang Chinese Medical University, Hangzhou, China; 4 College of Life Science, Zhejiang Chinese Medical University, Hangzhou, China; University of Texas Health Science Center at Houston, United States of America

## Abstract

Jieduquyuziyin prescription (JP), a traditional Chinese medicine (TCM) prescription, has been widely used for the clinical treatment of systemic lupus erythematosus (SLE). However, the complex chemical constituents of JP and the multifactorial pathogenesis of SLE make research on the therapeutic mechanism of JP in SLE challenging. In this paper, a serum metabolomics approach based on rapid resolution liquid chromatography coupled with quadrupole time-of-flight mass spectrometry (RRLC-Q-TOF/MS) was employed to acquire the metabolic characteristics of serum samples obtained from mice in the SLE model group, JP-treated group, prednisone acetate (PA)-treated group and control group. The orthogonal partial least squares (OPLS) was applied to recognize metabolic patterns, and an obvious separation of groups was obtained. Thirteen metabolites, namely, phosphatidylethanolamine (PE 20:3), hepoxilin B3, lyso- phosphatidylethanolamine (lyso-PE 22:6), 12S-hydroxypentaenoic acid (12S-HEPE), traumatic acid, serotonin, platelet-activating factor (PAF), phosphatidylcholine (PC 20:5),eicosapentaenoic acid (EPA), 12(S)-hydroxyei- cosatetraenoic acid (12S-HETE), 14-hydroxy docosahexaenoic acid (14-HDOHE), lyso-phosphatidylcholine (lyso-PC 20:4), and indole acetaldehyde, were identified and characterized as differential metabolites involved in the pathogenesis of SLE. After treatment with JP, the relative content of 12(S)-HETE, PAF, 12(S)-HEPE, EPA, PE (20:3), Lyso-PE(22:6), and 14-HDOHE were effectively regulated, which suggested that the therapeutic effects of JP on SLE may involve regulating disturbances to the metabolism of unsaturated fatty acid, tryptophan and phospholipid. This research also demonstrated that metabolomics is a powerful tool for researching complex disease mechanisms and evaluating the mechanism of action of TCM.

## Introduction

Systemic lupus erythematosus (SLE), a complex systemic autoimmune disease, is a multifactorial process characterized by multi-system and multi-organ impairment. A commonly accepted viewpoint holds that uncontrolled lymphocyte autoreactivity and dysregulated production of auto-antibodies by B cells lead to formation of immune complexes that can precipitate in the organs and cause tissue damage [1]. The multifactorial process of SLE makes research on its pathogenesis and therapeutic approach challenging [2]. Glucocorticoids and immunosuppressors are effective in the clinical treatment of SLE but are accompanied by serious side effects, such as femoral head necrosis and hypertension [3].

As a refractory disease, SLE involves multiple mechanisms, which suggests that traditional Chinese medicine (TCM), being based on holistic concepts and systems thinking, may have a good therapeutic effect. TCM is a treasure of Chinese civilization and has experienced a history of thousands of years of clinical practice. Jieduquyuziyin prescription (JP), which contains *Radix Paeoniae Rubra, Radix Rehmanniae*, *Carapax Trionycis, Radix Glycyrrhizae*, et al, is widely used for SLE treatment in China and has a good therapeutic effect on SLE [4]. More importantly, JP has fewer side effects than glucocorticoids and immunosuppressors, even after long-term use [5]. However, the complexity of the chemical constituents of JP makes study of its efficacy challenging.

Metabolomics is considered “the quantitative measurement of the dynamic multi-parametric metabolic response of living systems to pathophysiological stimuli or genetic modification” [6], and is also an important part of systems biology. Metabolomics studies the body from a holistic perspective, which coincides with the theory of TCM [7]. This specialty of metabolomics not only helps to reveal the scientific basis of TCM, but is also beneficial to understanding the pathogenesis and diagnosis of disease, as well as toxicological research [8]. Serum is commonly and effectively used as an object of metabolomic analysis because the serum metabolome is rich, comprising various classes of important biomolecules [9], [10]. Mass spectrometry (MS)-based metabolomics is well suited for reliably coping with high-throughput samples with respect to both technical accuracy and the identification and quantitation of low-molecular-weight metabolites which is primary concern of metabolomics [11]. Rapid resolution liquid chromatography coupled with quadrupole time-of-flight mass spectrometry (RRLC-Q-TOF/MS) is widely used for metabolomics studies because of its outstanding dynamic range and high sensitivity, which means that even low-response metabolites can be collected and identified [12]. In addition, MS and MS/MS information on metabolites acquired from RRLC-Q-TOF/MS are helpful for metabolite identification.

Mice treated with a single intraperitoneal injection of the pristane develop a lupus-like disease characterized by the production of autoantibodies directed against many lupus autoantigens [13], [14]; and with in-depth understanding of the mechanisms of pristane-induced SLE model, which are associated with IFN-I dysregulation, the mice model has been thought to highly relevant to human SLE [15]. In this paper, a RRLC-Q-TOF/MS-based metabolomics method was established to study the metabolic profiles and differential metabolites associated with SLE by analyzing serum specimens collected from the SLE model group, prednisone acetate (PA)-treated group, JP-treated group, and control group. Furthermore, some active compounds of JP were quantified by a rapid resolution liquid chromatography system coupled with triple quadrupole mass spectrometry (RRLC-QQQ/MS). This study may facilitate understanding of the pathological changes in SLE and the therapeutic mechanism of JP.

## Experimental Methods

### Chemicals and reagents

An HPLC grade of formic acid and acetonitrile were purchased from Tedia (Fairfield, OH, USA). Prednisone acetate tablets were purchased from Lisheng (Tianjin, China). Water was purified by a Milli-Q water purification system (Millipore, Bedford, MA, USA). Reference and calibration solutions were purchased from Agilent (Agilent, Santa Clara, CA, USA). Standards of catalpol, paeoniflorin, ferulic acid, liquiritin, rutin, hesperidin, quercetin, asiaticoside and glycyrrhizic acid were purchased from the National Institutes for Food and Drug Control, China.

### Raw herbal medicines and JP extract

The following raw herbal medicines were purchased from the Chinese Herbal Medicine Co. Ltd. of Zhejiang Chinese Medical University (Hangzhou, China). JP was a combination of dried root of *Radix Rehmanniae*, *Carapax Trionycis, Herba Artemisiae Annuae, Rhizoma Cimicifugae foetidae, Herba Hedyotidis, Radix Paeoniae Rubra, Herba Centellae Asiaticae, Semen Coicis, Fructus Citri Sarcodactylis* and *Radix Glycyrrhizae* (at ratios of 5∶4∶4∶9∶5∶4∶5∶5∶3∶2). Next, 120 g JP was cut into small pieces and soaked in water (1/10 *w/v*) for 1 h, then boiled for 2 h. The filtrate was collected and the residue was extracted again with the same volume of water for another 2 h. The filtrates were combined and concentrated under vacuum to make a 1200 mL volume of JP.

### Quantitative analysis of active compounds of JP

Some active compounds of JP, including catalpol, paeoniflorin, hesperidin, asiaticoside, glycyrrhetinic acid, quercetin, rutin and ferulic acid were quantified. The standards were precisely weighed and dissolved in methanol to plot the calibration curve of the standards. The extracted 100 mL JP was freeze-dried. Ultrasonic waves were used to help dissolve the residue in 20 mL methanol, and then the solution was filtered through 0.22 µm nylon filters.

JP was analyzed on an Agilent 1290 RRLC coupled to a 6460 QQQ/MS system with an ESI (Electron spray ionization) source (Agilent Technologies, Santa Clara, CA, USA). Samples were separated on an Agilent Eclipse Plus C18 column (2.1×50 mm, 1.8 µm) using 0.1% formic acid in water (A) and 0.1% formic acid in acetonitrile (B). The sample glass vials and column temperature were maintained at 4°C and 35°C, respectively. The gradient elution program was 5% B at 0 to 3 min, 5% to 15% B at 3 to 12 min, maintenance at 15% B for 6 min, 15% to 65% B at 18 to 35 min, 65% to 95% B at 35 to 39 min, and maintenance at 95% B for 2 min. The flow rate was kept constant at 0.3 mL min^−1^, and the post time was set at 4 min.

The parameters of the ESI source were optimized. The negative ion mode was applied; nebulizer gas was set at 35 psig, capillary voltage at 3,500 V, drying gas (N_2_) flow rate and temperature at 9 L min^−1^ and 350°C, respectively. The specific optimized MS and MS/MS parameters of the standards are shown in Table 1.

**Table 1:** The MS and MS/MS parameters of the standards and their quantitative results.

Name	No.	Retention time (min)	Fragmentor voltages (V)	Productor ion (m/z)	Collision energy (eV)	Product ion(m/z)	Content (µg/mL)	CV (%) (n = 5)	Accuracy%
**Catalpol**	1	0.66	120	(M+COOH)-:407.1	7	199.0, 361.0	4.07± 0.12	2.9%	97.4%
**Paeoniflorin**	2	10.91	120	(M+COOH)-:525.0	8	449.0, 121.0	230.15± 6.54	2.8%	102.4%
**Ferulic acid**	3	12.87	135	(M-H)-:193.0	12	133.8, 177.7	14.72± 0.45	3.0%	101.5%
**Liquiritin**	4	13.97	150	(M-H)-:417.1	15	254.9	6.58± 0.12	1.8%	98.2%
**Rutin**	5	14.96	150	(M-H)-:609.0	32	299.9	13.73± 0.51	3.7%	99.1%
**Hesperidin**	6	18.72	150	(M-H)-:609.1	20	300.9	10.13± 0.18	1.7%	103.4%
**Quercetin**	7	24.384	150	(M-H)-:301.0	20	150.9, 120.9	15.86± 0.64	4.0%	98.5%
**Asiaticoside**	8	25.67	150	(M+COOH)-:1003.4	18	957.3, 469.0	26.12± 1.04	3.9%	104.2%
**Glycyrrhizic acid**	9	28.98	160	(M-H)-:821.3	46	351.2, 193.1	120.66±3.63	3.0%	97.8%

### Ethics statement

All procedures were conducted in accordance to Animal Care and Use Committee guidelines of the Zhejiang Chinese Medical University. All methods were approved by the Institutional Animal Care and Use Committee of Zhejiang Chinese Medical University (Permission number: SYXK-ZHE-2008-0115).

### Animals and treatments

40 female C57BL/6J mice, 8 weeks old and weighing 22 to 26 g, were purchased from the Experimental Animal Center of Zhejiang Chinese Medical University and were housed in four cages. The mice were housed in a specific-pathogen-free (SPF) environment and had free access to a standard diet and tap water. All animals were kept in an animal room with suitable conditions: temperature at 20 ± 2°C, relative humidity at 55% ± 10%, and 12 h dark-to-light cycle. All animals were allowed to acclimate for 1 week before the experiment. The mice were randomly divided into four groups (n = 10): JP-treated group, PA-treated group, SLE model group, and control group; and JP-treated group, PA-treated group, SLE model group were given a single 0.5 mL intraperitoneal injection of pristine (Sigma-Aldrich, Louis, MO, USA). JP and PA were given orally (daily) to JP-treated group and PA-treated group, respectively, from 1 month after pristane injection and continued till 5 months [16]. PA was dissolved in physiological saline as a concentration of 0.69 mg mL^−1^, and the dosage was 18 mL kg^−1^ per day. The dosage of JP was also 18 mL kg^−1^ per day, and the SLE model group and control group were administered orally with an equivalent volume of physiological saline.

### RRLC-Q-TOF/MS analysis of metabolic profiling

After 10 weeks of treatment, blood samples were collected from the abdominal aorta to centrifuge tubes. Then 30 min later, the samples were centrifuged at 3,000 rpm for 10 min. Serum was transferred into other tubes and stored at −80°C until analysis.

Serum samples were thawed at room temperature, and then 450 µL acetonitrile was added to 150 µL serum and vortex-mixed for 1 min. The mixture was placed at 4°C for 10 min, and then centrifuged at 13,000 rpm for 10 min at 4°C. The supernatant was filtered through 0.22 µm nylon filter film and transferred to an autosampler vial, after which 4 µL of the sample was injected into the column.

Serum samples were analyzed on an Agilent 1260 RRLC coupled to a 6520 Q-TOF/MS system with a dual ESI source (Agilent Technologies, Santa Clara, CA, USA). Serum samples were separated on an Agilent Eclipse Plus C18 column (2.1×50 mm, 1.8 µm). The column temperature was set at 35°C and the flow rate was 0.3 mL min^−1^. The optimal mobile phase consisted of water with 0.1% formic acid (A) and acetonitrile with 0.1% formic acid (B). From 0 to 2 min, mobile phase B was maintained at 20%, from 2 to 12 min increased linearly from 20% to 65%, then increased to 95% in the next 13 min and kept at 95% for 3 min. The post time was set at 4 min. The sample glass vials were maintained at 4°C. The drying gas (N_2_) had a flow rate of 9 L min^−1^ and a temperature of 350°C. The positive and negative ionization modes were applied to acquire MS data, and the capillary voltages were at 4,000 V and 3,500 V, respectively. The skimmer voltage was set at 65 V and the fragmentor voltage at 135 V. MS data was acquired in full-scan mode from m/z 50 to 1,000 at the rate of 2 spectra s^−1^, and the collision energy of MS/MS data acquisition was set at 20 eV. The reference solution was continuously introduced into the MS system during analysis as an internal calibration of the Q-TOF system.

### Data analysis

The raw data were analyzed by an Agilent Mass Hunter (version 4.0 Qualitative Analysis, Agilent). The molecular feature extraction (MFE) algorithm was applied to extract metabolic features including *m/z,* retention time, and ion intensities from the total ion current (TIC) chromatograms. The main parameters of MFE were optimized as follows: the range of *m/z* values was 50 to 1000, and the thresholds of peak filters and compound filters set at 300 counts and 1000 counts, respectively. The abundance of each compound was defined as the summation of the isotopic peaks, adduct ion peaks and base peak of the compound. The results of MFE were imported into Mass Profiler Professional (MPP) software (version B 12.00, Agilent) for further data filtering, which included alignment, normalization, median-centering, and filtering by frequency. Firstly, the compounds with abundance greater than 3000 were aligned by accurate mass and retention time; the tolerance windows of mass and retention time were 10 ppm and 0.3 min, respectively. Secondly, the abundance of compounds was normalized and media-centered. Thirdly, missing peaks were filtered according to their frequency, and compounds that appeared in more than 80% of samples in at least one group were retained. The final compounds were exported to an Excel table (Microsoft, Redmond, USA). OPLS of SIMCA-P (Umetrics AB, Umea, Sweden) was applied to give a comprehensive view of clustering trends for the multidimensional data [17].

## Results and Discussion

### Quantitative analysis of active compounds in JP

**Figure 1:**
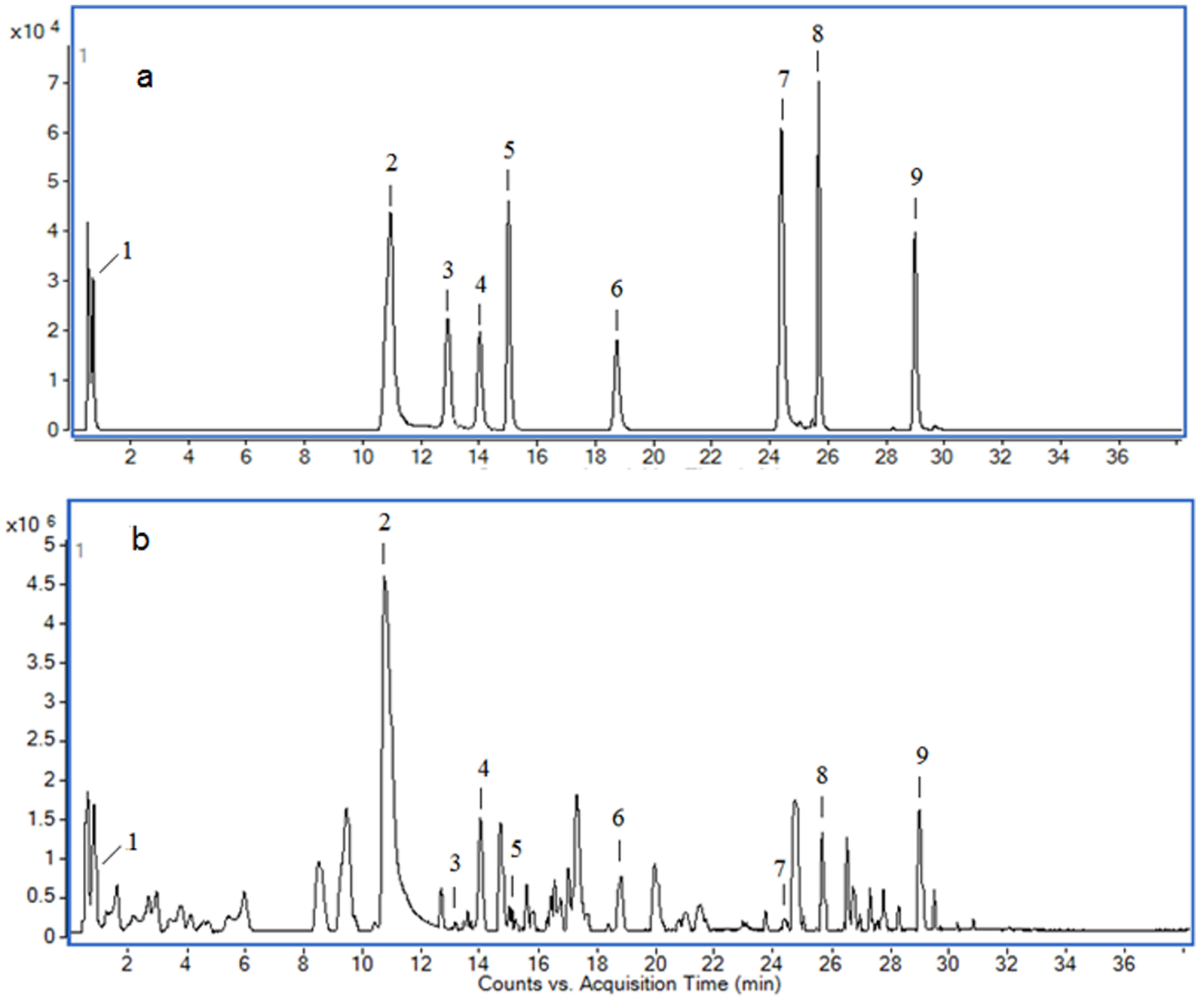
BPC of standards (a) and JP (b).

Base peak chromatogram (BPC) of the standards (Fig. 1A) and JP (Fig. 1B) were acquired. Calibration curves for the standards were plotted as the abundance of product ions versus concentration, and all r^2^ values were more than 0.994. The coefficient of variation (CV) and accuracy of the calibration curves, combined with the quantitative results are represented in Table 1.

### Method development and validation

The total protein concentration of blood serum is 60 to 80 g L^−1^ [18]; the high protein content of serum necessitates protein removal, for which the most commonly used method is protein precipitation with an organic solvent [19]. The optimal volume ratio of sample to acetonitrile was tested and used for protein precipitation [20]; a ratio of 1∶3 was employed, according to our usual practice. For the method validation study, 50 µL of serum the samples from each of the four groups were pooled to get a quality control (QC)specimen, and preparation of the QC specimen was the same as the samples. A number of consecutive injections of the QC sample were made to obtain a stable Q-TOF/MS system before experimental data acquisition, and then acquisition of data for the serum samples was started. QC specimens were analyzed every ten specimens throughout the whole analysis procedure.

For the QC sample, five characteristic ions (252.1336, 308.2324, 184.1462, 445.1456, 514.3085) were picked out to examine the drift of retention times, m/z and peak areas. The results showed that variations in the retention times were less than 0.17 min, drift values of m/z were less than 6 ppm and the relative standard deviation (RSD) of each peak area was below 8%, demonstrating that the system had excellent stability and repeatability during the analysis procedure.

### Metabolic profile of SLE

**Figure 2:**
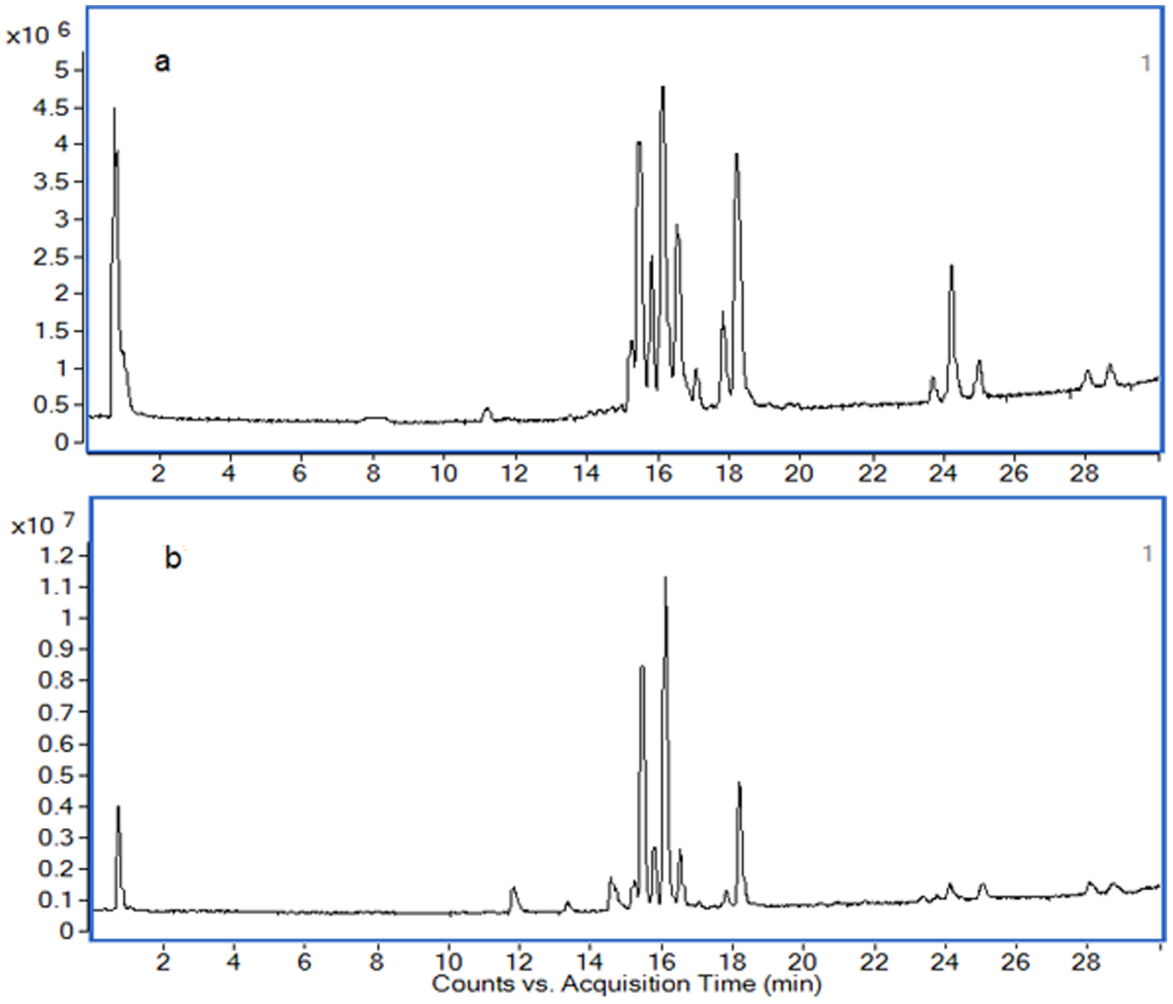
Typical TIC chromatograms for serum obtained in the positive ion mode (a) and negative ion mode (b).

**Figure 3:**
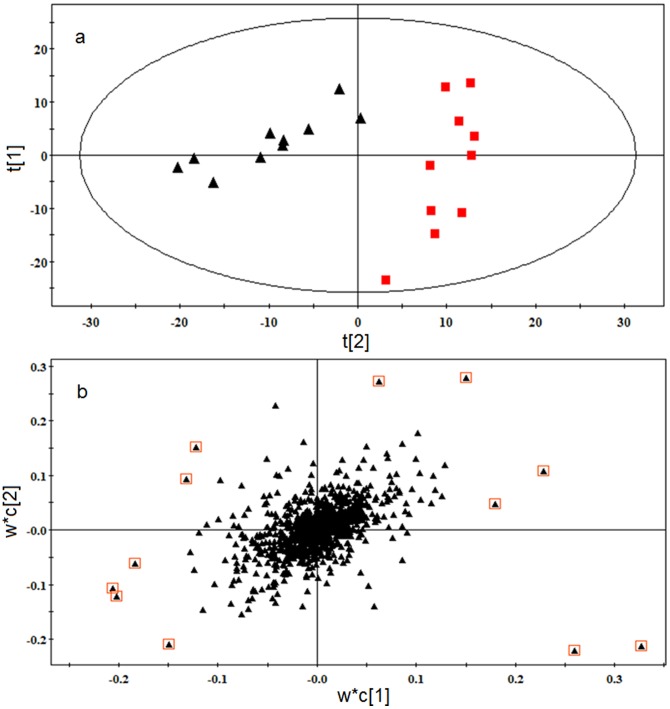
(a) OPLS score plot of the SLE model group (▴) and control group (▪). (b) OPLS loading plot of the SLE model group and control group. The 12 metabolites far from the origin that contributed significantly to differentiating the clustering of the SLE model group from the control group were defined as differential metabolites.

The typical TIC chromatograms of serum were acquired in positive ionization mode (Fig. 2A) and negative ionization mode (Fig. 2B). Alignment, normalization, median-centering, and filtering by frequency of MPP were applied to process the data, and finally 948 compounds of the positive and negative ion modes were obtained. Multivariate statistical analysis can give a comprehensive view of the clustering trend for complex metabolomics data [21]. The OPLS of SIMCA-P was used for multivariate analysis, and the CV-ANOVA (Analysis of variance testing of cross-validated residuals) was performed to validate the OPLS model. The p-value of CV-ANOVA was far lower than 0.01, and those results interpreted that the OPLS model was a significant model. A OPLS score plot base on all 948 compounds using 3 components (R^2^X = 0.536, R^2^Y = 0.931 and Q^2^ = 0.917) showed a obvious separation of the control group and the SLE model group(Fig. 3A). SLE model mice deviated from control mice in their metabolic profile, indicating that the metabolic networks of SLE mice were disordered. The differential metabolites associated with SLE were found according to the loading plot of OPLS (Fig. 3B) and the p-value of student's t-test.

**Figure 4:**
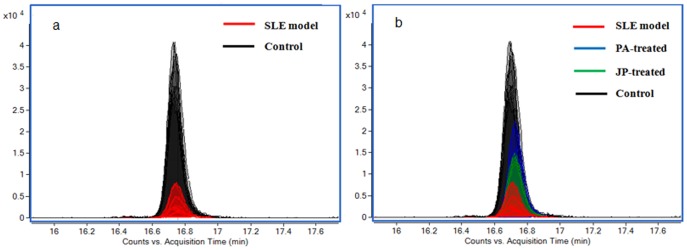
(a) Merged EIC of 14-HDOHE based on the SLE model group and control group. The relative content of 14-HDOHE significantly increased in SLE model mice. (b) Merged EIC of 14-HDOHE based on SLE model group, control group, JP-treated group, and PA-treated group.

**Figure 5:**
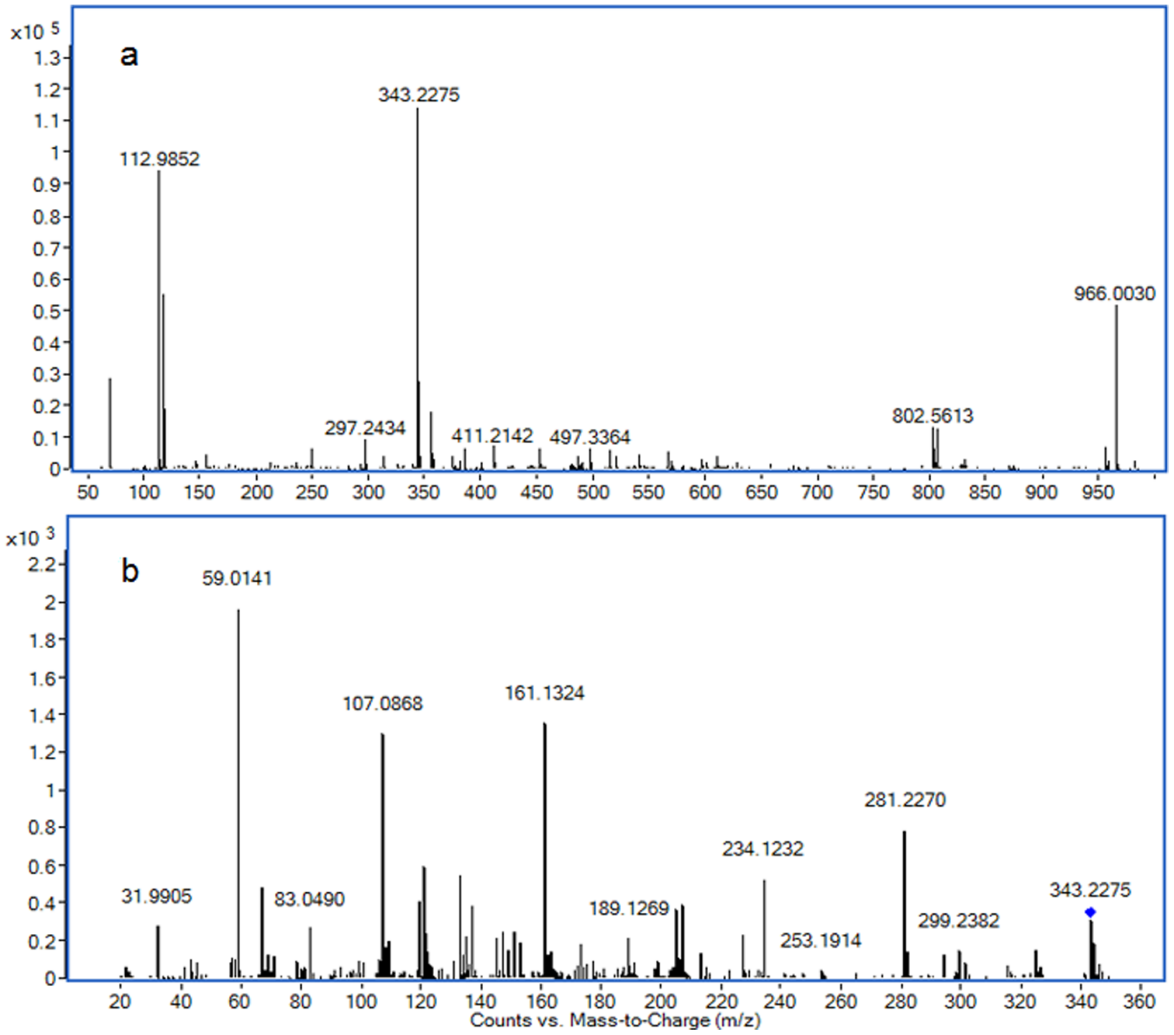
MS (a) and MS/MS (b) information of 14-HDOHE.

Differential metabolites were identified according to their MS and MS/MS information. The *m/z* 343.2275 was taken to illustrate the process of differential metabolite identification. Firstly, according to data analysis results of differential metabolites, the relative content of *m/z* 343.2275 was significantly different, and was determined as an [M-H]- ion by its MFE and MS information. Secondly, the extracted ion chromatograms (EIC) of *m/z* 343.2275 of all samples were obtained, and these chromatograms were merged (Fig. 4). The merged EIC visual reflects the relative content change of metabolites among groups and confirms that a suitable statistical method was used for data analysis. Thirdly, the MS/MS information about the fragmentation pattern of *m/z* 343.2275 was acquired from the Q-TOF system with 20 eV of collision energy. The following main fragment ions were acquired: 59.0141, 107.0868, 161.1324, 121.0645, 133.1018 (Fig. 5). The METLIN database was searched for the MS/MS information [22], and *m/z* 343.2275 was identified as 14-hydroxy docosahexaenoic acid (14-HDOHE). The identification process of other differential metabolites was similar to that of 14-HDOHE; the identification results and their trends of change among groups are shown in table 2.

**Table 2:** Identification and trends of change for differential metabolites.

Mass	Retention time (min)	Acquisition Mode	Name	Formula	Metabolic pathway	Trend in SLE model group a	Trend in JP-treated group b	Trend in PA-treated group b
**159.0682**	0.77	ESI+	Indole acetaldehyde	C_10_H_9_NO	Tryptophan metabolism	↓^**^	↑	↑
**176.0948**	0.78	ESI+	Serotonin	C_10_H_12_N_2_O	Tryptophan metabolism	↓^**^	↓	↑
**228.1361**	12.21	ESI+	Traumatic acid	C_12_H_20_O4	α- Linolenic acid metabolism	↑^**^	↓	↓^*^
**302.2245**	16.18	ESI+	EPA	C_20_H_30_O_2_	Fatty acid biosynthesis	↓^**^	↑^**^	↑^**^
**523.3637**	17.45	ESI+	PAF	C_26_H_54_NO_7_P	Phosphatidic metabolism	↑^**^	↓^*^	↓^**^
**543.3331**	15.45	ESI+	Lyso-PC(20:4)	C_28_H_50_NO_7_P	Phospholipid metabolism	↓^**^	—	—
**541.3162**	14.66	ESI+	PC (20:5)	C_28_H_48_NO_7_P	Phospholipid metabolism	↓^**^	—	—
**503.2992**	18.05	ESI+	PE (20:3)	C_25_H_46_NO_7_P	Phospholipid metabolism	↑^*^	↓^*^	↓^*^
**320.2354**	17.85	ESI-	12(S)-HETE	C_20_H_32_O_3_	Arachidonic acid metabolism	↑^**^	↓^**^	↓^*^
**318.2195**	16.19	ESI-	12(S)-HEPE	C_20_H_30_O_3_	EPA metabolism	↓^**^	↑^**^	↑^*^
**344.2353**	17.53	ESI-	14-HDOHE	C_22_H_32_O_3_	DHA metabolism	↓**	↑*	↑**
**336.2304**	16.43	ESI-	Hepoxilin B3	C_20_H_32_O_4_	Arachidonic acid metabolism	↓^**^	↑	↑
**525.2857**	15.38	ESI-	Lyso-PE(22:6)	C_27_H_44_NO_7_P	Phospholipid metabolism	↑^*^	↓^*^	↓^*^

a Change trend compared with control group.

b Change trend compared with SLE model group.

The levels of differential metabolites were marked with (↓) down-regulated, (↑) up-regulated and (—) no significant change (*P<0.05; **P<0.01).

### Therapeutic effect of JP on SLE

**Figure 6:**
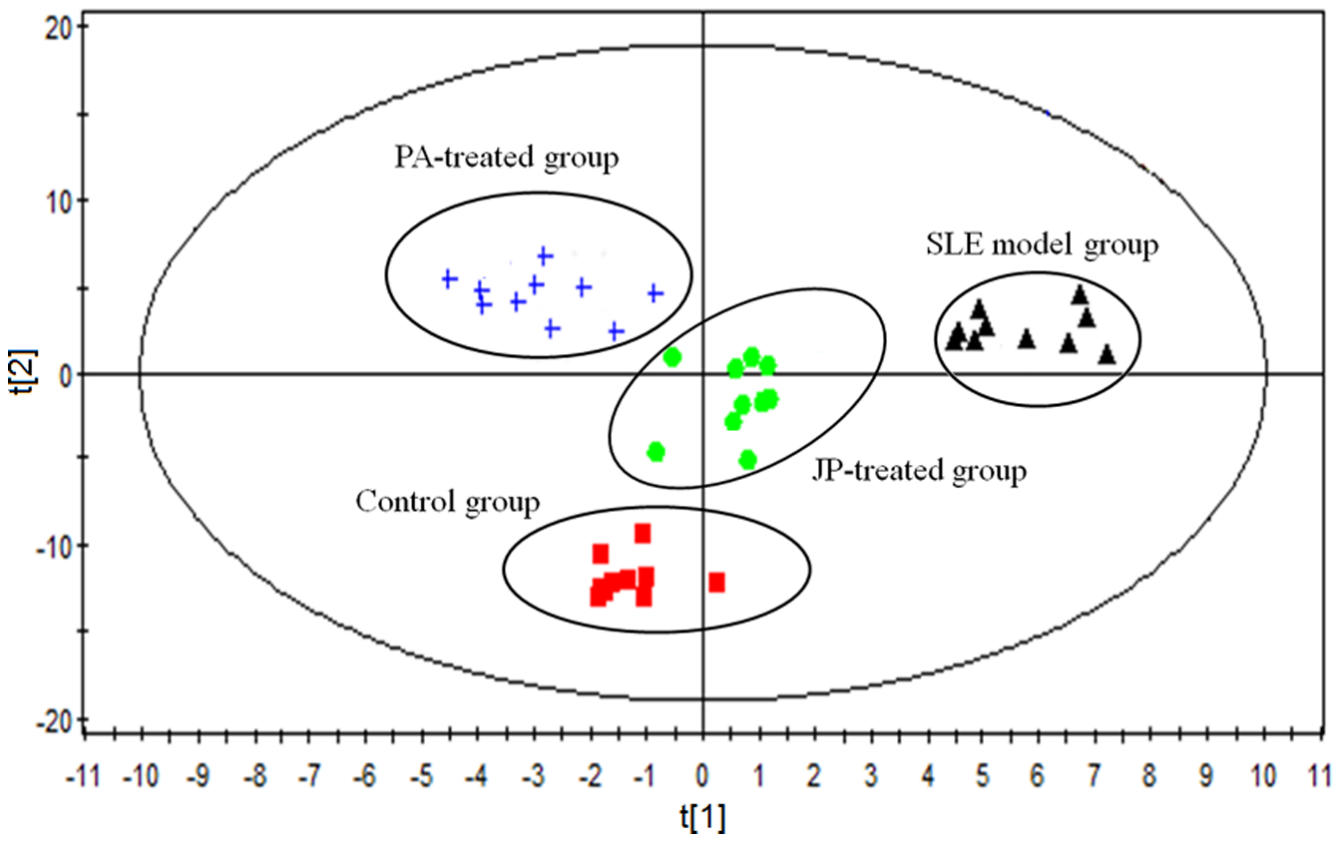
OPLS score plot of the SLE model group, PA-treated group, JP-treated group and control group by SIMCA-P11.0 (n = 10 in each group).

The results of differential metabolite identification suggest that the development of SLE involves serious disorders of the metabolism of unsaturated fatty acids (UFAs), phospholipid and tryptamine. The dysfunction of the immune system in SLE mice may be caused by metabolic disorders. Disorders of UFA metabolism, especially of arachidonic acid (AA), eicosapentaenoic acid (EPA), and docosahexaenoic acid (DHA), probably aggravate the multi-organ and multi-system inflammatory response, thus promoting the development of SLE. After treatment with JP or PA, the score plot (R^2^X = 0.489, R^2^Y = 0.894 and Q^2^ = 0.786) of OPLS (Fig. 6) showed that there was a significant difference in the metabolic profile of the SLE model group, PA-treated group, JP-treated group and control group. This difference confirmed that JP and PA can play a role in regulating the abnormal metabolic network of SLE mice. PA could effectively regulate the relative content of differential metabolites such as traumatic acid, due to its anti-inflammatory and immunosuppressive function. JP, which exerts a multi-pathway and multi-target role in the body, effectively regulated the metabolic disorders of mice with SLE. The relative content of EPA, 12S-hydroxyeicosatetraenoic acid (12S-HETE), 14-HDOHE, 12(S)-HEPE, and Platelet-activating factor (PAF) in the JP-treated group were prominently regulated, and the content of these differential metabolites trended to the level of the control group (Table 2). JP effectively repaired the UFA metabolic network, thus alleviating the adverse effects of differential metabolites on immune response and inflammation, ameliorating the dysfunction of the immune system and the multi-organ inflammatory response during the development of SLE, thereby inhibiting the progression of SLE.

### Biochemical interpretation of differential metabolites

**Figure 7:**
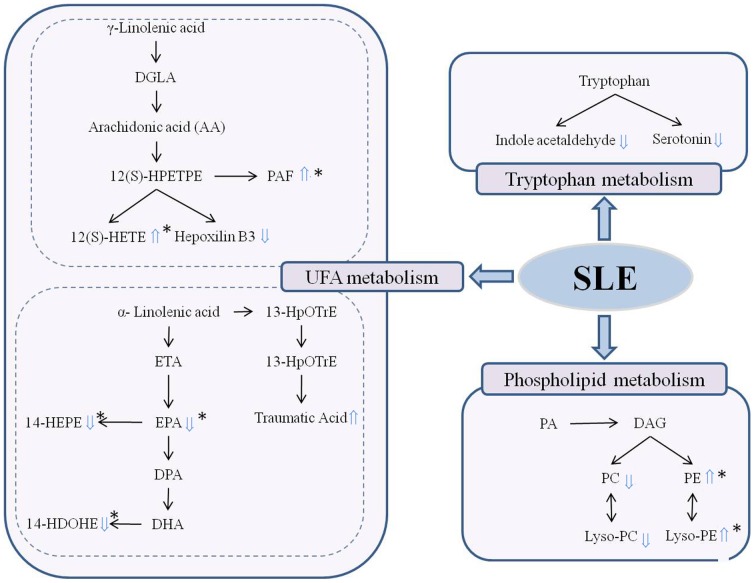
The perturbed metabolic network associated with SLE. The differential metabolite levels of the SLE model group compared to the control group were marked with (⇑) upregulated and (⇓) downregulated. (*Differential metabolites which could be effectively regulated by JP).

Fig. 7 shows the perturbed metabolic network associated with SLE. The metabolic pathways of UFAs, tryptophan and phospholipid were involved in the pathogenesis of SLE. UFAs perform an important role in maintaining normal physiological function, including regulation of the immune response. Deficiency of UFA can accentuate or improve the symptoms of certain autoimmune diseases in animals [23]. In this experiment, multiple metabolites of UFA metabolic pathways were involved in the pathogenesis of SLE. The relative content of 14-HDOHE, EPA and its active metabolite 12S-hydroxypentaenoic acid (12S-HEPE) were decreased in the serum of the SLE model group, while the content of 12(S)-HETE, and traumatic acid were elevated. These results indicate that UFA metabolism is involved in the development of SLE. 12(S)-HEPE originating from 12-lipoxygenase (12-LOX) oxidation of EPA is thought to elicit an inhibitory effect on platelet aggregation. DHA and EPA metabolism produce a variety of metabolites; these weaker physiologically active metabolites competitively inhibit the biosynthesis of inflammatory mediators such as leukotrienes B_4_ (LTB_4_) and prostaglandin E_2_ (PGE_2_), and have an anti-inflammatory efficacy [24]. Metabolic disturbances to EPA and DHA may affect their ability to regulate the immune response, thereby increasing the symptoms of SLE. 12(S)-HETE is an active metabolite of arachidonic acid produced through the 12-lipoxygenase pathway. It has a powerful role in promoting inflammation and causes the accumulation of extracellular matrix and induces mesangial cell hypertrophy via p38 MAPK [25]. The role of 12(S)-HETE in the pathogenesis of diabetic nephropathy has been confirmed [26]. Increased 12(S)-HETE may promote kidney inflammation and cell hypertrophy when SLE occurs, thereby aggravating the symptoms of kidney disease. UFA metabolic disorders would result in a low serum level of anti-inflammatory metabolites and a high serum level of pro-inflammatory metabolites; these abnormal alterations may be one factor to explain why the pathogenesis of SLE is accompanied by a systemic inflammatory response.

Moreover, there is evidence indicating that perturbations of intestinal microbiota composition or function may play an important role in the development of diseases associated with altered metabolism [27], and that the intestinal microbiota can regulate the absorption, metabolism and storage of host lipid and UFAs via numerous microbial activities [28], [29]. Therefore, the immune system disorder associated with SLE may be related to the intestinal microbiota dysfunction that adversely affects UFA metabolism. On the other hand, the dysbiosis of intestinal microbiota also can directly perturb the immune regulatory networks [30]. The close links between the intestinal microbiota, the immune system and lipid metabolism inspires an idea that incorporates intestinal microbial function into an in-depth study of the prominent disorders of UFA metabolism and the immune system in SLE model mice, as well as the possibility of treating SLE by regulating the intestinal microbiota.

Phospholipid metabolites, including phosphatidylcholine (PC 20:5), Lyso-PC (20:4), phosphatidylethanolamine (PE 20:3), Lyso-PE(22:6) and PAF were identified as the differential metabolites of SLE. The phospholipids are important precursors for many biologically active mediators of metabolism including eicosanoids, diacylglycerol and PAF. PEs also play important roles in biological processes such as apoptosis and cell signaling [31]. The abnormal phospholipid metabolic pathway may not only result in the abnormal of physiology and metabolism via a variety of pathways, but also promote the systemic inflammatory state [32]. PAF, a highly bioactive inflammatory phospholipid mediator, plays an important role in the pathogenesis of allergic reactions, acute and chronic inflammatory reactions, and arthritis [33]. PAF is also believed to play a role in various syndromes or diseases, including renal diseases, by favoring immune complex formation and modulating the subsequent inflammatory reaction [34]. PAF-induced proteinuria could be prevented by a PAF receptor antagonist, which further illustrates the physiological role of PAF in glomerular permeability [35]. In this study, SLE model mice showed obviously elevated serum levels of PAF, which is probably related to the serious lupus nephritis. PAF can promote platelet aggregation and can also stimulate the generation of auto-antibodies by activating platelets. In brief, activated platelets aggregated with circulating antigen-presenting cells, including monocytes and plasmacytoid dendritic cells, thus stimulating significant auto-antibody [36]. Therefore, PAF may become a new target for the treatment of SLE: PAF enzymes or PAF receptor antagonists may restrain the effects of PAF action, thereby inhibiting the proinflammatory and platelet activation it causes [37].

Indole acetaldehyde and serotonin are tryptophan metabolites, whose content declined in the SLE model group. Tryptophan has a very sensitive regulation function for the T cell cycle and can promote the conversion of T cells in the spleen [38]. However, tryptophan is susceptible to the influence of kidney disease and immune system disorders leading to metabolic disorders [39]. The tryptophan metabolic pathway was significantly affected by SLE, and disorder of tryptophan metabolism is also been reported in MRL/lpr mice, a spontaneous SLE model, in our previous study [40]. Therefore, the tryptophan metabolism is susceptible in process of SLE, and the abnormal tryptophan metabolism can influence the apoptosis of T cell [41], and impact the development of SLE.

## Conclusion

RRLC-Q-TOF/MS-based serum metabolomics analysis combined with multivariate statistical analysis was applied to evaluate the pathogenesis of SLE and the mechanism of action of JP in SLE model mice. There was a prominent metabolic profile difference between SLE model mice and control mice according to OPLS. Thirteen differential metabolites in the SLE model were identified by the MS and MS/MS information. These differential metabolites confirm that SLE is associated with specific abnormalities in the metabolism of UFAs, tryptophan and phospholipid. JP can effectively regulate the UFA and phospholipid metabolic pathway and exert a good therapeutic effect on SLE. These results provide a better understanding of the pathogenesis of SLE and the mechanism of action of JP in SLE. Our results also prove that metabolomics is a powerful technology platform for studying the mechanism of action of TCM and investigating disease pathogenesis.
